# Acute duodenal obstruction secondary to intussusception caused by the duodenal diverticulum: a case report

**DOI:** 10.1186/s12876-020-01379-9

**Published:** 2020-07-22

**Authors:** Yuchen Guo, Bin Liu, Ziwen Pan, Yang Zhang

**Affiliations:** Department of Gastrointestinal Surgery, First Hospital of Jilin University, Changchun, 130021 Jilin China

**Keywords:** Duodenal diverticulum, Intussusception, Endoscopy, Duodenal obstruction, Case report

## Abstract

**Background:**

The duodenal intussusception is rarely reported and usually occurs secondary to organic diseases of the duodenum such as polyps, tumors and duplication cysts. Herein we report a case of duodenal intussusception caused by duodenal diverticulum.

**Case presentation:**

A 21-year old male patient presented with abdominal pain and vomiting for one day. A contrast enhanced computed tomography of the abdomen revealed duodenal intussusception. On emergency laparotomy, the intussusception had reduced spontaneously while an invaginated diverticulum was seen at the junction of the descending and horizontal segments of the duodenum. The diverticulum was resected and the patient had uneventful recovery.

**Conclusion:**

Duodenal intussusception is a rare complication of duodenal diverticulum. Being aware of this complication of diverticulum can help in timely diagnosis and treatment.

## Background

Intussusception is characterized by telescoping of the proximal bowel loop into the distal bowel. Primary idiopathic small bowel intussusception is common in children while secondary intussusception is usually present in adults with intestinal diseases such as tumor, polyps, tubercles, adhesions and Meckel diverticulum. However, the intussusception of duodenum is rarely reported. Herein, we report a case of duodenal diverticulum that invaginated in the duodenal lumen causing intussusception and obstruction. Very few such cases have been reported in the English literature [1].

## Case presentation

**Fig. 1:**
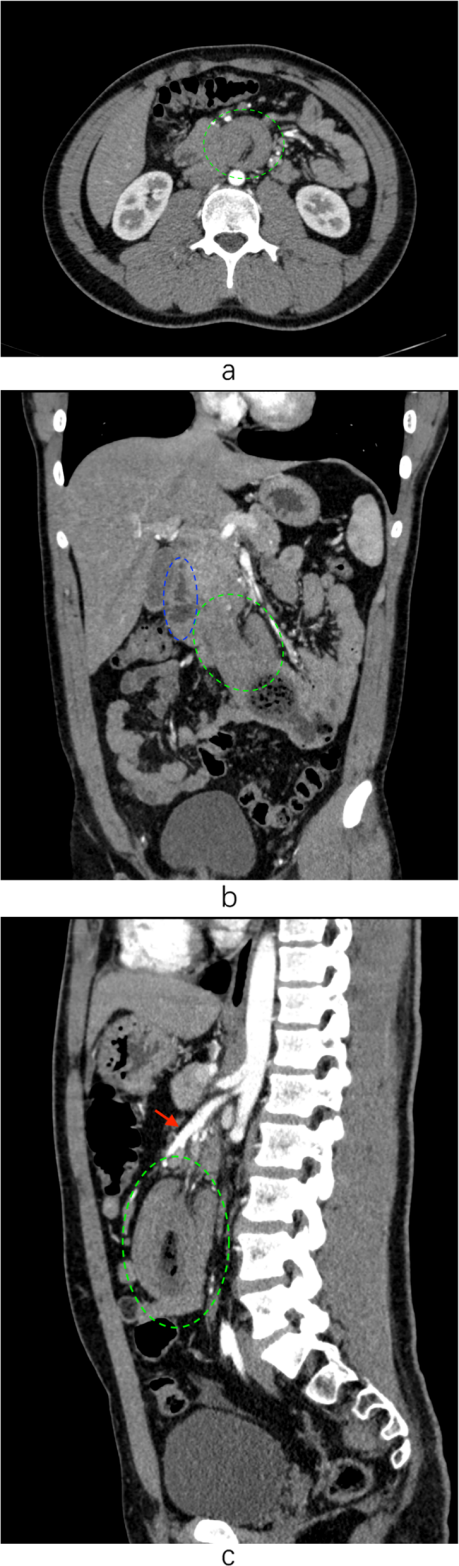
**a**. The cross section of abdominal CT shows the typical bowel-within-bowel sign suggestive of duodenal intussusception into the jejunum (green circle). The intestinal wall of the head was thickened. The degree of strengthening was slightly reduced. Lumpy contents can be seen in the duodenal cavity. **b.** The coronal section of the CT scan shows the intussusception of duodenum into the jejunum (green circle) and the descending part of duodenum (blue circle) **c.** The sagittal section of the CT scan shows the intussusception (green circle) and its positional relation with superior mesenteric artery (red arrow)

**Fig. 2:**
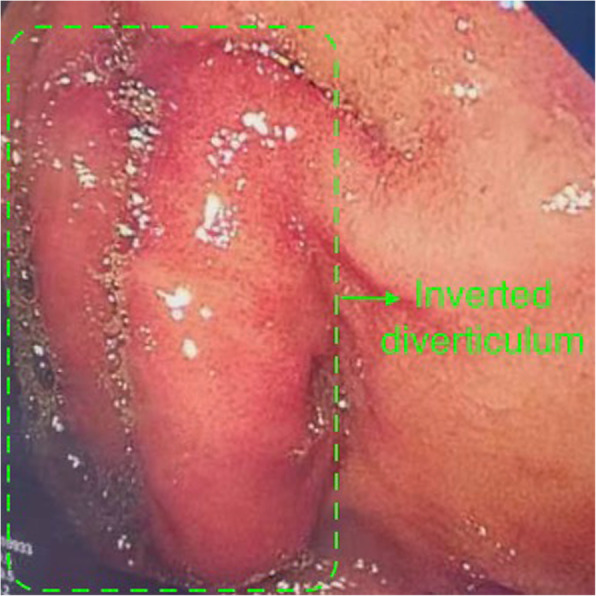
A diverticulum was seen at the junction of the descending and horizontal segments of the duodenum, which had turned inward into the lumen of the duodenum (green arrow)

**Fig. 3:**
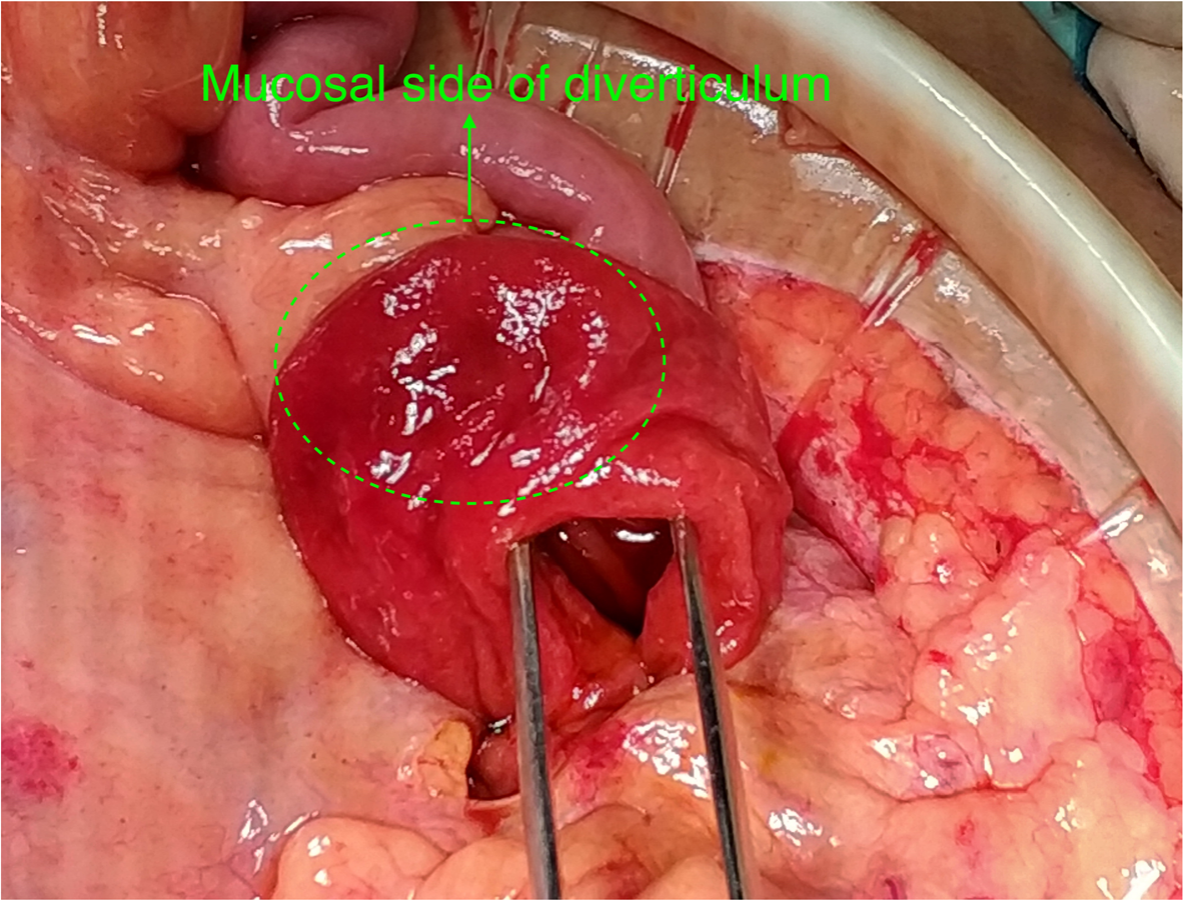
Duodenal lumen was opened at the base of the diverticulum. The diverticulum was turned inside out. The green arrow indicates the mucosal side of the diverticulum

**Fig. 4:**
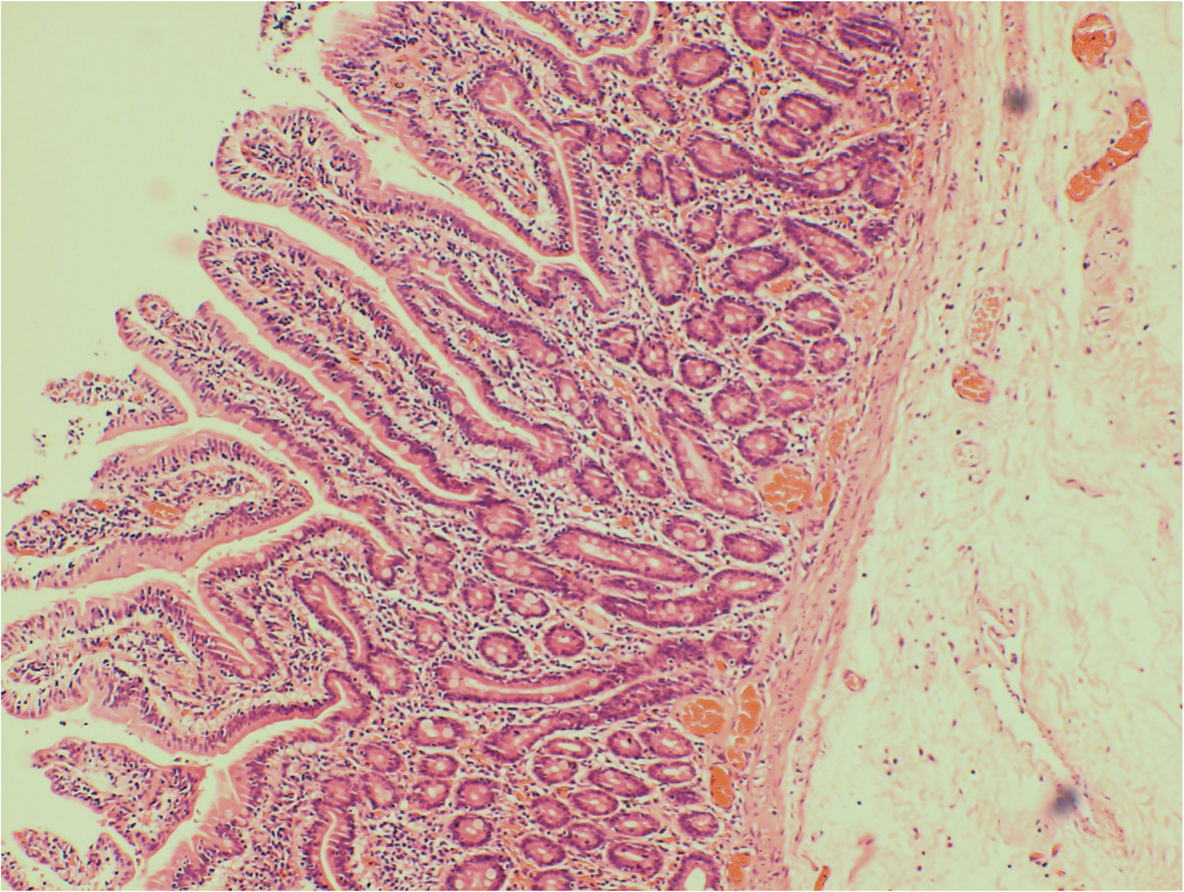
The histopathological examination of the resected diverticulum revealed submucosal edema, vasodilatation, congestion and hemorrhage. Additionally, acute and chronic inflammatory cell infiltration was also seen

A 21-year old male presented with severe intermittent abdominal pain, accompanied by vomiting for one day. Physical examination was unremarkable. During the admission, he underwent contrast enhanced computed tomography (CT) of the abdomen which revealed a duodeno-jejunal intussusception (Fig. 1a-c). Presence of intestinal tumor or polyp could not be excluded. We could not perform a gastroscopy because of the severe symptoms of upper gastrointestinal obstruction of this patient. So, decision to perform exploratory laparotomy was taken. During the operation, the horizontal and descending segments of the duodenum were found to be dilated. However, no obvious intussusception or intestinal lesion was observed during the operation. So, we performed intraoperative gastroscopy via oral route. A large diverticulum was seen at the junction of the descending and horizontal segments of the duodenum, which had invaginated into the lumen of the duodenum (Fig. 2). Considering it to the lead point of intussusception, we planned the surgical excision of the diverticulum. We made incision at the base of the diverticulum, resected the duodenal diverticulum and sutured the duodenal incision (Fig. 3). The histopathological report of the resected specimen indicated presence of submucosal edema, vasodilatation, congestion and hemorrhage. Acute and chronic inflammatory cell infiltration was also present (Fig. 4). The postoperative recovery was uneventful with the postoperative hospital stay of 8 days. At one-year follow-up, the patient is symptom-free.

## Discussion and conclusions

Bowel intussusception is rare in adults, accounting for 5% of the intussusceptions in all age group and 1–5% cases of bowel obstruction in adults [2]. Among them, duodenal intussusception is extremely rare. Duodenal intussusception can occur because of the excessive mobility of the duodenal wall in cases with intestinal malrotation [3]. Duodenal intussusception without intestinal malrotation is usually not seen due to the relatively fixed retroperitoneal position of the duodenum. There are very few reports of the duodenal intussusception caused by various factors such as prolapse of duodenal tumors, ampullary lesions, duplication cysts, and congenital malrotation [3–6]. However, in the present case, duodenal intussusception occurred due to diverticulum. In extreme cases, duodenal intussusception can lead to biliary obstruction [7, 8].

Duodenum is the second most frequent site in the digestive tract for diverticular disease. Duodenal diverticulum mostly occurs in the second or third portion of the duodenum along the pancreatic or mesenteric border, and commonly near the ampulla of Vater [9]. Duodenal diverticula can be congenital or, more frequently, an acquired pseudodiverticula. They are usually asymptomatic. Approximately 5% of them are associated with complications, such as hemorrhage, obstruction, compression of biliopancreatic structures, inflammation and perforation. In the current case, the duodenal diverticulum got invaginated into the lumen of the duodenum and got pushed into the proximal jejunum due to intestinal peristalsis leading to intussusception. However, we didn’t observe the intussusception during the surgery due to spontaneous reduction, as reported in previous cases [10, 11]. Duodenal intussusception is generally transient and non-obstructive [11]. Sometimes the duodenal intussusception may retrograde spontaneously because of the poor mobility of the duodenal wall [10].

The clinical manifestations in adults with duodenal intussusception are usually non-specific and include nausea, vomiting and epigastric pain [6]. Abdominal CT is a very sensitive modality for diagnosis and can usually detect the lead point responsible for intussusception if present. CT may reveal the typical bowel-within-bowel sign. However, mucosal prolapse can mimic these signs in the absence of intussusception [12]. Some cases of duodenal intussusception have been reported, but it is unclear whether this phenomenon is true intussusception or simple mucosal prolapse, which is misinterpreted as intussusception [3, 13, 14]. However, in the present case, the diverticulum had invaginated into the lumen of the duodenum, which acted as the lead point of the intussusception. So, we believe that the present case didn’t had simple mucosal prolapse but intussusception. Endoscopy is another useful imaging modality for the diagnosis of intussusception and its lead point if present. Additionally, it is useful in making tissue diagnosis which helps in planning definitive treatment. Also, endoscopy may help reduce the duodenal intussusception before surgery. We believe that retrograde traction may occur due to gastric and proximal duodenal dilatation due to insufflation. However, this hypothesis needs to be proven by future studies. The management of duodenal intussusception depends on the underlying cause and severity of symptoms. If the cause is malignancy then pancreatoduodenectomy is required. It is a benign disease such as adenoma or polyp or diverticulum as seen in the present case then simple endoscopic or surgical excision of the lesion is curative. Some cases of intestinal malrotation precipitating duodenal intussusception may also require surgical correction.

Duodenal intussusception secondary to duodenal diverticulum is rarely reported. Duodenal intussusception should be considered in patients with duodenal diverticulum having persistent or recurrent abdominal symptoms. Detailed investigations should be performed to make the correct diagnosis. Endoscopy may help reduce the duodenal intussusception before surgery due to gastric and proximal duodenal dilatation due to insufflation. However, this hypothesis needs to be proven by future studies.

## Data Availability

Not applicable.

## Abbreviation

CT: Computed tomography
